# Low colonization rates with Multidrug-resistant Gram-negative bacteria in a German hospital-affiliated hemodialysis center

**DOI:** 10.1371/journal.pone.0240314

**Published:** 2020-10-15

**Authors:** Ralph Wendt, Olaf Nickel, Almut Botsch, Margareta Lindner, Angela Bethge, Kathrin Marx, Bernhard R. Ruf, Joachim Beige, Christoph Lübbert

**Affiliations:** 1 Department of Infectious Diseases/Tropical Medicine, Nephrology and Rheumatology, Hospital St. Georg, Leipzig, Germany; 2 Kuratorium for Dialysis and Transplantation (KfH) Renal Unit, Hospital St. Georg, Leipzig, Germany; 3 Department of Laboratory Medicine and Microbiology, Hospital St. Georg, Leipzig, Germany; 4 Hospital Pharmacy, Hospital St. Georg, Leipzig, Germany; 5 Martin-Luther-University Halle/Wittenberg, Halle, Germany; 6 Division of Infectious Diseases and Tropical Medicine, Department of Oncology, Gastroenterology, Hepatology, Pneumology and Infectious Diseases, Leipzig University Hospital, Leipzig, Germany; 7 Interdisciplinary Center for Infectious Diseases, Leipzig University Hospital, Leipzig, Germany; Universita degli Studi della Campania Luigi Vanvitelli, ITALY

## Abstract

**Background:**

Multidrug-resistant Gram-negative bacteria (MDRGN) are found with rising prevalence in non-hemodialysis risk populations as well as hemodialysis (HD) cohorts in Asia, Europe and North America. At the same time, colonization and consecutive infections with such pathogens may increase mortality and morbidity of affected individuals. We aimed to monitor intestinal MDRGN colonization in a yet not investigated German HD population.

**Methods:**

We performed cross-sectional point-prevalence testing with 12 months follow-up and selected testing of relatives in an out-patient HD cohort of n = 77 patients by using microbiological cultures from fresh stool samples, combined with Matrix Assisted Laser Desorption Ionization—Time of Flight Mass Spectrometry (MALDI-TOF-MS) and antimicrobial susceptibility testing.

**Results:**

We detected MDRGN in 8 out of 77 patients (10.4%) and 1 out of 22 relatives (4.5%), indicating only colonization and no infections. At follow-up, 2 patients showed phenotypic persistence of MDRGN colonization, and in 6 other patients de-novo MDRGN colonization could be demonstrated. Pathogens included *Escherichia coli* and *Klebsiella pneumoniae* (with extended-spectrum beta-lactamase [ESBL]-production as well as fluoroquinolone resistance), *Stenotrophomonas maltophilia* and *Enterobacter cloacae*.

**Conclusions:**

In a single-center study, MDRGN colonization rates were below those found in non-HD high-risk populations and HD units in the US, respectively. Reasons for this could be high hygiene standards and a strict antibiotic stewardship policy with evidence of low consumption of fluoroquinolones and carbapenems in our HD unit and the affiliated hospital.

## Introduction

Infections with antibiotic-resistant bacteria are a striking problem in patients with chronic diseases and compromised immune status. Among patients who require chronic hemodialysis (HD), Vancomycin-resistant enterococci (VRE) and methicillin-resistant *Staphylococcus aureus* (MRSA) have been studied extensively [1–3] and showed important differences of Gram-positive bacteria distribution between European and other regions [4]. Other concerns have been raised in terms of spreading multidrug-resistant Gram-negative bacteria (MDRGN) among non-HD chronic-disease patient populations [1, 5, 6] since these pathogens are associated with up to five times higher mortality rates compared with susceptible Gram-negative bacteria regarding blood stream infections [7, 8]. In Western Europe, colonization and infection with MDRGN is emerging especially in healthcare-associated facilities such as nursing homes [9, 10] and intensive care units (ICUs) reaching colonization rates up to 53% [5]. In other parts of Europe and the world, MDRGN are even more widely distributed in the community setting [11–13] and among travelers [12, 14, 15].

Mortality among end-stage renal disease (ESRD) patients is multifold higher compared to the general population [16] with infections being the second most common reason to die [17]. Apparently, infection patterns are changing nowadays, with MRSA infections constituting a lower proportion compared to the rise of MDRGN infections [1] in both non-HD populations and presumably HD patients [6]. For instance, in the Boston area of the US in 2008 a MDRGN colonization rate of 27% was recorded in a large cohort of HD out-patients [18]. In a British HD center from 2011 to 2014, 84 patients experienced 95 Gram-negative infections [19]. Despite these trends, antimicrobial resistance among Gram-negative bacteria has not been previously investigated in chronic HD patients in Europe or Germany. Hence, data on the distribution of MDRGN in HD out-patients cohorts are scarce and the association between colonization and the risk for infections is not yet clear.

We therefore performed a cross-sectional point-prevalence study with sampling follow-up to determine the presence of MDRGN among out-patients who require chronic HD in our center in Leipzig, Germany.

## Patients and methods

### Study setting

Patients from a single HD unit were included in a prospective cohort study analyzing the period May 1, 2015 to June 30, 2017 after providing voluntary written informed consent. The out-patient unit services nephrological care for 167 patients and is affiliated with a large 1060-bed municipal hospital in Eastern Germany. Among the patients, 140 were able to consent and 77 agreed to participate (55%). Fifty-five of these 77 patients (71%) took part in a follow-up investigation 12 months later, and relatives (household members) of 22 patients gave consent as well and were tested following the same procedure. The reasons for non-participation after 12 months were: 10 patients died, 9 patients rejected participation, and 3 patients were transplanted or had recovering kidney function. Trained nurses in the HD unit performed the collection of specimens for MDRGN testing by fecal sampling. All patients underwent 4-hour hemodialysis three times a week.

### Microbiological approach

**Fig 1:**
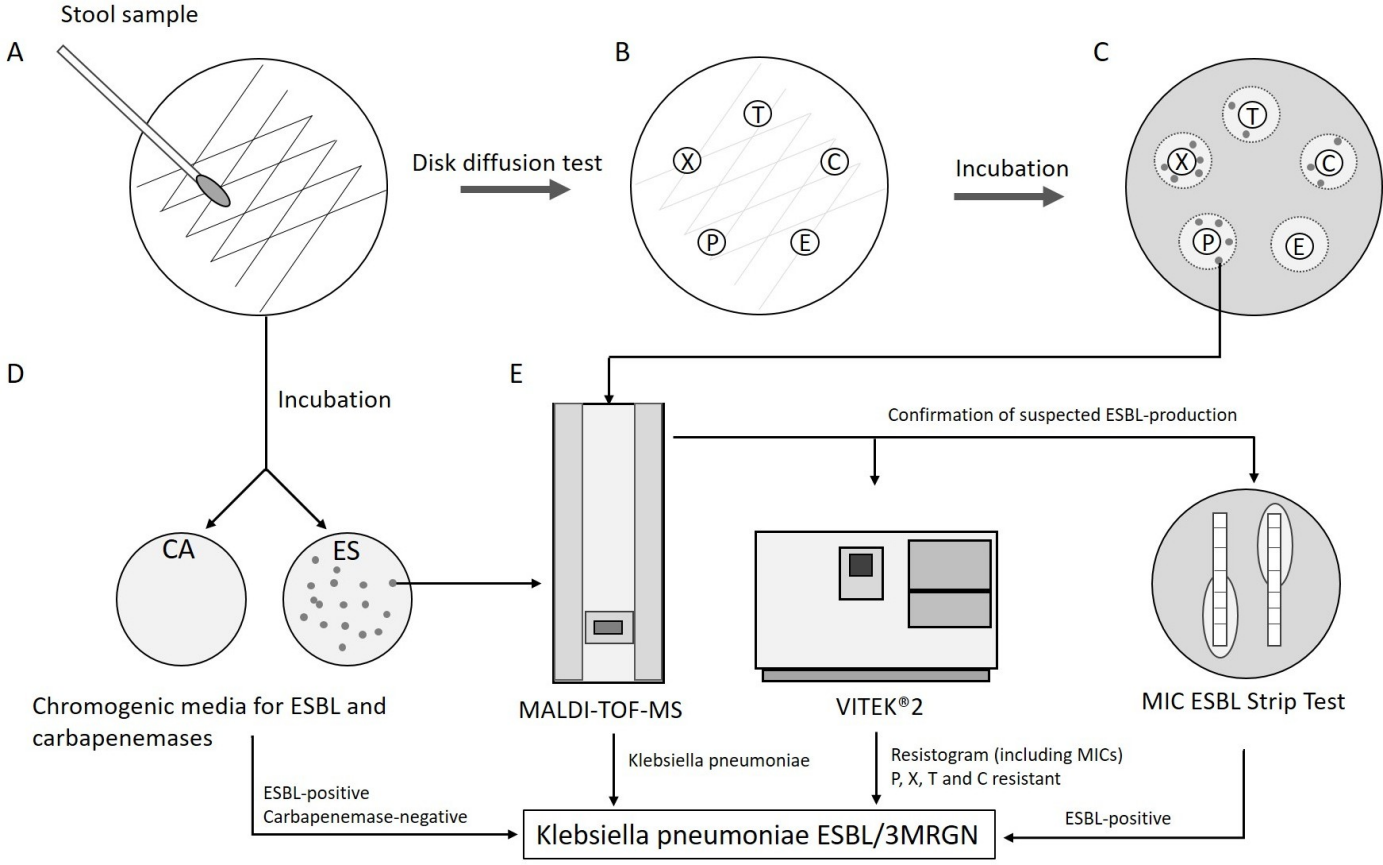
**(A)** Inoculation of stool samples on blood agar, endo agar, and chromogenic agar plates for ESBL and carbapenemase producers (ES and CA). **(B)** Application of antimicrobial susceptibility disks for piperacillin (P), cefotaxime (X), ceftazidime (T), ciprofloxacin (C) or ertapenem (E) to blood and endo agar plates. **(C)** After 24 hours of incubation at 36°C one colony of each phenotype (grey spots) within the inhibition zones was isolated. **(D)** In parallel, colonies growing on selective chromogenic media were isolated. **(E)** The isolated strains were identified by MALDI-TOF-MS and susceptibility testing was performed by microdilution using the VITEK®2 system. Semi-automated MIC determination was performed using CLSI breakpoints. Suspected ESBL production within the isolated strains was confirmed by MIC strip ESBL testing.

Specimens were primarily investigated by conventional microbiological cultures. Therefore, fresh stool samples were spread on non-selective Columbia blood agar, Gram-negative selective endo agar and chromogenic selective media (ChromID® ESBL agar, bioMérieux, Nürtingen, Germany, and Brilliance™ CRE medium, Oxoid, Wesel, Germany) (**Fig 1A**). Subsequently, commercially available antimicrobial susceptibility disks were applied to blood and endo agar plates (Becton Dickinson, Heidelberg, Germany) (**Fig 1B**). If adequate inhibitory zones were absent in the agar diffusion test, one representative colony of each phenotype was isolated from the area surrounding the antimicrobial disks for piperacillin, cefotaxime, ceftazidime, ciprofloxacin and ertapenem (**Fig 1C).** In parallel, colonies that grew on selective chromogenic media for extended spectrum beta-lactamases (ESBL) or carbapenemases were isolated accordingly (**Fig 1D**). Bacterial species from these isolates were identified by VITEK®-MS (bioMérieux, Nürtingen, Germany) using Matrix Assisted Laser Desorption Ionization—Time of Flight Mass Spectrometry (MALDI-TOF-MS) (**Fig 1E**).

Determination of minimum inhibitory concentrations (MIC) was carried out semi-automatically using the VITEK®2 system and Advanced Expert System™ software (AES) (bioMérieux, Nürtingen, Germany) according to manufacturer’s instructions, employing Clinical and Laboratory Standards Institute (CLSI) breakpoints (**Fig 1E**) [20]. Strains producing ESBLs were confirmed by using MIC Test Strip ESBL technology (Cefotaxime / Cefotaxime + Clavulanic acid; Ceftazidime / Ceftazidime + Clavulanic acid; Liofilchem, Roseta, USA) (**Fig 1E**). Additional resistance against fluoroquinolones was confirmed by VITEK®2-AST-N289/N248 cards (bioMérieux, Nürtingen, Germany). Classification of MDRGN was done according to the German national guideline (MDRGN are henceforth designated “**M**ulti-**R**esistant **G**ram-**N**egatives”, MRGN, with the subtypes 3MRGN and 4MRGN, as summarized in **Table 1**) [21, 22].

All patients were informed about their test results by the supervising nephrologist.

**Table 1:** Classification of MDRGN according to the German national guideline [21].

Bacteria	Categories^1^				Classification	
	I	II	III	IV	3MRGN	4MRGN
Enterobacterales^2^	PIP	TAX or TAZ	CIP	ETP or IMI or MER or CARB	Resistance to three out of four categories	Resistance to four out of four categories or to category IV alone

^1^PIP = piperacillin, TAX = cefotaxime, TAZ = ceftazidime, CIP = ciprofloxacin, ETP = ertapenem, IMI = imipenem, MER = meropenem, CARB = carbapenemase detected in the isolate irrespectively of the resistance phenotype, intermediate test results are considered as resistant for the classification.

^2^Enterobacterales includes a classification for the following species: E. coli, Klebsiella spp., Proteus spp., Citrobacter spp., Enterobacter spp., Serratia marcescens, Morganella spp., Providencia spp.

### Ethics approval

This work complies with ethical regulation for human studies and was conducted in accordance with the World Medical Association Declaration of Helsinki (1964) and its later amendments. Approval from the local ethics committee (Ethics Committee of the Saxonian Board of Physicians, Dresden, Germany) was obtained before the start of the study, specifically before data access and sample collection (EK-BR 52-15/1).

### Statistics

Descriptive statistics of patient data with pseudonymized ID numbers were performed with standard procedures using MS Excel^®^ (Microsoft, Redmond, Washington, USA).

## Results

Out of a total of 77 patients, 2 tested initially positive for ESBL-producing *Escherichia (E*.*) coli* with fluoroquinolone co-resistance (3MRGN) (2.6%), 5 tested positive for *E*. *coli* with ESBL-production (6.5%), and 1 patient tested positive for *Stenotrophomonas maltophilia*. In 22 available samples from relatives, ESBL-producing *E*. *coli* with fluoroquinolone co-resistance (3MRGN) were observed in 1 person, but the resistance phenotype differed from that of the HD patient. No patient suffered from any illness related to MDRGN colonization during the observation period.

At follow-up after 12 months, 1 patient remained positive for ESBL-producing *E*. *coli* with fluoroquinolone co-resistance (3MRGN), 1 patient remained positive for *E*. *coli* with ESBL-production, and 6 other patients had evidence of de-novo colonization with MDRGN. One of these patients acquired an ESBL-producing *E*. *coli* strain with fluoroquinolone co-resistance (3MRGN) between initial testing and follow-up, 3 patients acquired an *E*. *coli* strain with ESBL-production, and 2 patients acquired either multi-drug resistant *Klebsiella pneumoniae* or *Enterobacter cloacae* strains. Out of 5 patients with initial evidence of *E*. *coli* with ESBL-production, 4 showed spontaneous decolonization at follow-up.

The overall positivity rate of potentially pathogenic Gram-negative bacteria in all samples (initial, follow-up and relatives, n = 154) was 10.4%. Concerning tested patients, any colonization observed in either initial or follow-up sampling was noted in 14 out of 77 patients (18.1%). Among the pathogens, ESBL-producing *E*. *coli* constituted the most frequent finding, including 2 isolates with co-resistance to fluoroquinolones (3MRGN). No carbapenem-resistant isolates were detected. Colonization did not impact either morbidity or mortality within a follow-up period of 12 months.

## Discussion

To our knowledge, this study is the first to investigate both follow-up sampling as well as MDRGN colonization rates of patients’ relatives in a HD setting. The observed colonization rates are far below those given in the literature in non-HD high risk populations such as nursing homes [6, 9] and HD units in the US [18], respectively. However, the applied methodology for MDRGN screening differs from other approaches cited in the literature [6, 9, 18]: Hogardt et al. exclusively used selective ESBL CHROMagar as primary screening media [9], and O’Fallon et al. and Pop-Vicas et al. relied on selective MacConkey agar containing low concentrations of fluoroquinolones and ceftazidime for screening [6, 18]. We believe that the combination of an agar diffusion test and two MDRGN selective media variants is probably more sensitive than a single selective media approach. Hence, it seems more likely that the observed low MRDGN colonization rates in our study are due to substantial differences in basic hygiene standards or (true) epidemiological reasons. Obviously, with a colonization rate of ESBL-producing *E*. *coli* with fluoroquinolone co-resistance (3MRGN) of approximately 3% and a complete absence of carbapenem-resistant bacteria, the background epidemiological pressure in our setting is not as high as it has been described in the literature.

Regarding antibiotic routine regimes both in out-patient dialysis as well as in the affiliated hospital, we sought to establish a robust and targeted conventional therapy for calculated initial and de-escalating therapy for Gram-negative infection with a wide variation and diversification of antibiotic usage, especially saving carbapenems [23, 24]. Compared to other large municipal hospitals in Germany we have a lower use of carbapenems and fluoroquinolones and a much higher stake of tetracyclines (primarily doxycycline), at least partially explaining the low 3MRGN colonization rate in our patients (**Table 2**) [25].

**Table 2:** Mean antibiotic application rates in recommended daily doses (RDD) per 100 hospital days in our inpatient nephrology ward including out- and inpatient dialysis unit during 2015, 2016 and 2017 in comparison to the average application rate of the 25% lowest using hospital sectors in Germany (right outer column) in the same time period [25].

	2015	2016	2017	Average consumption rate in the lowest-using 25% quartile of German hospitals (2015–2017)
	RDD/100 hospital days			
Fluoroquinolones	2.38	2.95	3.28	5.3
Third generation cephalosporines	2.48	3.89	3.98	2.9
Carbapenems	0	0.06	0.24	1.6
All antibiotics	4.86	6.91	7.5	9.8

Future research should aim to target microbiological resistance profiles in HD populations under conditions of different antibiotic policies. In this respect, longitudinal investigations using serial testing for the appearance and disappearance of MDRGN colonization may help to understand environment–host interactions including selection pressure by antibiotics. A related issue affects the role of contacts between nursing personnel, in-patients and out-patients in HD units and the underlying compliance with hygiene standards, especially hand hygiene.

### Limitations

Taking into account reports of cases testing MDRGN-negative for a period of time and converting again, possibly related to interrupted intestinal shedding of MDRGN, some authors suggest a series of at least 3 to 4 consecutive negative stool specimens, separated by sufficient time intervals (i.e., ≥1 week), before successful MDRGN decolonization is determined [26]. Moreover, we were unable to distinguish between persistence of MDRGN carriage and possible recolonization during HD stay as well as cross-transmission from relatives to HD patients and vice versa. Only the use of whole-genome sequencing might have allowed us to answer this question.

## Conclusion

In conclusion, the present study contradicts the belief that HD units are MDRGN hotspots. MDRGN carriage was lower than observed in a similar US study and in non-HD, but high-risk populations, and was not associated with MDRGN infections.
